# A question of Mazurov on groups of exponent dividing 12

**DOI:** 10.1080/00927872.2020.1788569

**Published:** 2020-07-08

**Authors:** Chimere Stanley Anabanti, Sarah Beatrice Hart, Michael C. Slattery

**Affiliations:** a Institut für Analysis und Zahlentheorie, Technische Universität Graz (TU Graz), Graz, Austria;; b Department of Economics, Mathematics and Statistics, Birkbeck, University of London, London, UK;; c Department of Mathematical and Statistical Sciences, Marquette University, Wisconsin, USA

**Keywords:** Exponent, finite presentation, groups, 20F05, 20D60

## Abstract

Mazurov asked whether a group of exponent dividing 12, which is generated by *x*, *y* and *z* subject to the relations $x^{3}=y^{2}=z^{2}={\left(xy\right)}^{3}={\left(yz\right)}^{3}=1,$ has order at most 12. We show that if such a group is finite, then the answer is yes.

The following question of Mazurov is listed as Question 19.53 in the collection of open problems in the Kourovka Notebook [2].

Question 1 (Mazurov). *Let G be a group of exponent 12 generated by elements x, y, z such that* $x^{3}=y^{2}=z^{2}={\left(xy\right)}^{3}={\left(yz\right)}^{3}=1$*. Is it true that* |G|≤12*?*

Recall that the ***exponent*** of a group *G* is the smallest positive integer *n* such that $g^{n}=1$ for all g∈G; meanwhile, *G* has *period n* whenever the exponent of *G* divides *n*. In fact, the question as stated in [2] requires “exponent 12” rather than “exponent dividing 12”, but Mazurov has confirmed to the authors that “exponent dividing 12” (that is, period 12) was intended. If the answer to the question is yes, then one consequence would be that groups of period 12 are locally finite (see [3]).

As a step in this direction, we have the following. Here, *C*_3_ is the cyclic group of order 3, and *A*_4_ is the alternating group of degree 4.

Lemma 2. *Let G be a group of exponent dividing 12, which is generated by x, y and z subject to the relations* $x^{3}=y^{2}=z^{2}={\left(xy\right)}^{3}={\left(yz\right)}^{3}=1$*. If G is finite, then G is either trivial or isomorphic to either C_3_ or A_4_.*

Proof. Let *G* be a group of exponent dividing 12 with the given presentation. Then *G* certainly satisfies the additional relations ${\left(xz\right)}^{12}=1$ and ${\left(\mathrm{xyz}\right)}^{12}=1.$ Therefore *G* is a quotient of the group *U* given by

$$U:=〈x,y,z|x^{3}=y^{2}=z^{2}={\left(xy\right)}^{3}={\left(yz\right)}^{3}={\left(xz\right)}^{12}={\left(\mathrm{xyz}\right)}^{12}=1〉.$$

We observe that a finite group *G* of exponent dividing 12 must have order $2^{a}3^{b}$ for some *a* and *b*. Therefore, by Burnside’s Theorem, *G* is solvable. Hence, *G* is a solvable quotient of *U*. We may therefore employ the command

SolvableQuotient(U);

in MAGMA [1]. This function returns the largest solvable quotient of a given finitely presented group. The outcome is as follows.

>U: = Group < x,y,z|x ⁁ 3, y ⁁ 2, z ⁁ 2,(x*y) ⁁ 3,(y * z) ⁁ 3, (x * z) ⁁ 12, (x * y * z) ⁁ 12>;>SolvableQuotient(U);GrpPC of order 12 = 2 ⁁ 2 * 3PC-Relations:$.1 ⁁ 3 = Id($),$.2 ⁁ 2 = Id($),$.3 ⁁ 2 = Id($),$.2 ⁁ $.1 = $.3,$.3 ⁁ $.1 = $.2 * $.3

Therefore, |G|≤12. It is now quick to check by hand that the only possibilities for G, apart from the trivial group, are $C_{3}\mathrm{and}A_{4}.$

We note that, in terms of the original Question 1 above, Lemma 2 shows that if a group of exponent exactly 12 with the given relations exists, then it must be infinite.
